# CircularRNA_104670 plays a critical role in intervertebral disc degeneration by functioning as a ceRNA

**DOI:** 10.1038/s12276-018-0125-y

**Published:** 2018-08-06

**Authors:** Jian Song, Hong-Li Wang, Ke-Han Song, Zhi-Wen Ding, Hai-Lian Wang, Xiao-Sheng Ma, Fei-Zhou Lu, Xin-Lei Xia, Ying-Wei Wang, Fei-Zou, Jian-Yuan Jiang

**Affiliations:** 1 Department of Orthopaedics, Huashan Hospital, Fudan University, Shanghai, China 200040; 2 Shanghai Institute of Cardiovascular Diseases, Zhongshan Hospital, Fudan University, Shanghai, China 200032; 3 Department of Anesthesiology, Huashan Hospital, Fudan University, Shanghai, China 200040

## Abstract

This study was carried out to explore the roles of circular RNAs (circRNAs) in nucleus pulposus (NP) tissues in intervertebral disc degeneration (IDD). Differentially expressed circRNAs in IDD and normal NP tissues were identified based on the results of microarray analysis. Bioinformatics techniques were employed to predict the direct interactions of selected circRNAs, microRNAs (miR), and mRNAs. CircRNA_104670 was selected as the target circRNA due to its large multiplier expression in IDD tissues. After luciferase reporter and EGFP/RFP reporter assays, we confirmed that circRNA_104670 directly bound to miR-17-3p, while MMP-2 was the direct target of miR-17-3p. The receiver-operating characteristic (ROC) curve showed that circRNA_104670 and miR-17-3p had good diagnostic significance for IDD (AUC _circRNA_104670_ = 0.96; AUC _miRNA-17-3p_ = 0.91). A significant correlation was detected between the Pfirrmann grade and expression of circRNA_104670 (*r* = 0.63; *p* = 0.00) and miR-17-3p (*r* = −0.62; *p* = 0.00). Flow-cytometric analysis and the MTT assay showed that interfering with circRNA_104670 using small interfering RNA (siRNA) inhibited NP cell apoptosis (*p* < 0.01), and this inhibition was reduced by interfering with miR-17-3p. Interfering with circRNA_104670 suppressed MMP-2 expression and increased extracellular matrix (ECM) formation, which were also reduced by interfering with miR-17-3p. Finally, an MRI evaluation showed that circRNA_104670 inhibition mice had a lower IDD grade compared with control mice (*p* < 0.01), whereas circRNA_104670 and miRNA-17-3p inhibition mice had a higher IDD grade compared with circRNA_104670 inhibition mice (*p* < 0.05). CircRNA_104670 is highly expressed in the NP tissues of IDD and acts as a ceRNA during NP degradation.

## Spinal disease: A roundabout route to disc degeneration

‘RNA sponges’ may provoke lower back pain by soaking up regulatory RNAs that normally protect the protein infrastructure surrounding cells in intervertebral discs. Many people suffer from lower back pain arising from disc degeneration (IDD). A team led by Fei-Zou and Jian-Yuan Jiang at Fudan University, Shanghai, China set out to identify molecular mechanisms that might contribute to IDD. They focused on circular RNAs, non-protein coding RNAs that have been linked to a variety of diseases. The researchers learned that IDD is associated with strongly elevated expression of a circular RNA that acts as an ‘RNA sponge’, binding to and thereby inactivating other RNA molecules. This inactivation ultimately results in the excessive production of an enzyme that can damage the protein matrix that supports cells within spinal discs, potentially setting up the conditions for IDD.

## Introduction

Increasing attention has focused on musculoskeletal disorders of the spine, which are associated with low back pain, morbidity, and mental disease. Several published studies have confirmed that intervertebral disc degeneration (IDD) is mainly responsible for musculoskeletal disorders of the spine^1–3^. Basal research has shown that the progression of IDD can be delayed or prevented by treating nucleus pulposus (NP) tissue at the molecular level since IDD has been shown to be involved in a variety of cellular events, from matrix synthesis to cytokine expression^4–8^. It is also important to clarify the regulation of extracellular matrix (ECM) catabolism during IDD as this process is always accompanied by the accumulation of ECM-degrading molecules and inflammatory mediators^9^.

Circular RNAs (circRNAs) are defined as a large class of non-coding RNAs (ncRNAs) that are composed of special exonic sequences in the absence of a free 3 or 5 end^10^. Increasing attention has been paid to circRNAs, as statistical estimates and biochemical assays provide a powerful confirmation that circRNAs exist as a significant portion of the spliced transcripts from hundreds of genes^11,12^. Hansen et al.^13^ specifically elucidated the mechanism of circRNAs by confirming that circRNAs may act as transcription regulators or as sponges for small RNA regulators, which compete for microRNA (miRNA) activity in the process of regulating cell metabolism.

It has been confirmed that circRNAs are involved in a larger number of diseases; however, the roles of circRNAs in the metabolic regulation of IDD are still unknown. Thus, the purpose of this study was to identify the circRNAs that are altered in IDD because these circRNAs may play a role in regulating IDD and may be potential targets in IDD therapy.

## Materials and methods

### Tissue specimens and cell culture

Twenty-nine NP specimens from 14 IDD and 15 normal subjects were enrolled in the study. NP specimens were collected from IDD patients suffering from cervical spondylotic myelopathy who received anterior cervical discectomy and fusion (ACDF), while NP specimens of normal subjects were obtained from patients with Hirayama disease who received ACDF at Huashan Hospital, Fudan University. None of the enrolled subjects had undergone radiotherapy or chemotherapy and none had a surgery history. Among the collected NP tissues, eight samples (eight samples from normal and IDD subjects) were used for microarray analysis of circRNAs, 20 samples (ten samples from normal and IDD subjects) were used for quantitative real-time PCR, and one sample was used for the functional verification of the selected circRNA. NP tissues were released from intervertebral disc (IVD) tissue and then incubated with 0.25 mg/mL type II collagenase (Jrdun bio, Shanghai, China) in Dulbecco’s modified Eagle medium (DMEM) (Jrdun bio, Shanghai, China) at 37 C for 12 h. After isolation, NP cells were cultured in DMEM supplemented with 2 mM glutamine, 10% fetal bovine serum (FBS), and 5 μg/mL gentamicin; the cultured NP cells were then maintained at 37 °C in a humidified atmosphere containing 5% CO2. No significant changes in morphology were observed between primary (passage0) and later-passage (passage2) cells; therefore, we used second-passage cells cultured in a monolayer for our experiments. All studies involving human subjects were approved by the appropriate review board(s) of the Ethics Committee of Huashan Hospital of Fudan University, and all of the studies abided by the Declaration of Helsinki principles. The study protocol was also approved by the Ethics Committee of Huashan Hospital.

### Microarray analysis of circRNAs

NP specimens from IDD and normal subjects were collected and immediately frozen in liquid nitrogen. Eight NP samples (four samples IDD and four normal subjects) were selected and homogenized in TRIzol reagent (Invitrogen, Carlsbad, CA, USA). We then quantified the total number of RNAs in each sample using a NanoDrop ND-1000. CircRNAs were enriched by removing linear RNAs with Rnase R (Epicentre, Madison, WI, USA). CircRNAs were then amplified and labeled with the Arraystar Super RNA Labeling Kit (Arraystar). After washing the slides, we scanned the arrays with an Agilent G2505C Scanner (Jamul, CA, USA). The acquired array images were analyzed via Agilent Feature Extraction software (version 11.0.1.1). For all circRNAs from NP specimens from IDD and normal subjects, fold-changes ⩾1.5 and *p* values < 0.05 were considered to be significantly different. All of the operation procedure was performed based on the protocols of Arraystar (Rockville, MD, USA), and Quantile normalization and subsequent data processing was performed using the R software package.

### Target prediction of ceRNA

An upregulated circRNA was selected based on microarray analysis of circRNAs. Quantitative real-time PCR was conducted to verify the selected circRNA. A circRNA- miRNA-mRNA network was constructed to predict the interaction between the selected circRNA, sponged miRNAs and target gene (mRNA) using miRNA target prediction software (Arraystars home-made) developed from TargetScan and miRanda. The triple network was finally built based on the ceRNA theory in which circRNA shares the same miRNA with mRNA or other ncRNAs in one triplet.

### Quantitative real-time PCR

Total RNA was isolated from NP cells from IDD and normal subjects using TRIzol reagent. CircRNA and mRNA quantification was conducted with the ABI PRISM7500 system, and the miRNA concentrations were also determined using the ABI PRISM7900 system (Applied Biosystems, Carlsbad, CA, USA). GRAPDH was used to normalize the relative expression levels of circRNA and mRNA, and the levels of small nuclear U6 were used to normalize the miRNA expression levels. The primers used for Real-time PCR are described below. CircRNA_104670: forward: 5’-GATGATCCTCTTCTCCAGCCAC-3’ and reverse: 5’-TGAAAGTAACCACAGCAACCAA-3’; Grapdh: forward: 5’- GGGAAACTGTGGCGTGAT - 3’ and reverse: 5’- GAGTGGGTGTCGCTGTTGA- 3’; MMP-2: forward: 5’- ACAACTTCTTCCCTCGCAAG - 3’ and reverse: 5’-ACAACTTCTTCCCTCGCAAG- 3’; miRNA-17-3p: forward: 5’- ACTGCAGTGAAGGCACTTGTAG- 3’; reverse: 5’- GGTCCAGTTTTTTTTTTTTTTTCTACA-3’.

### Diagnostic verification of the selected RNAs

Twenty NP tissues (ten samples from normal subjects and ten samples from IDD subjects) were used for quantitative real-time PCR, and the Pfirrmann grade was recorded for each tissue ^14^. The receiver-operating characteristic (ROC) curves of the circRNA and mRNA were used to diagnose IDD, and the area under the curve (AUC) was calculated. Correlation analysis was also carried out between the Pfirrmann grading and selected RNAs (circRNA and miRNA).

### Luciferase and EGFP/RFP reporter assay

We used the luciferase and EGFP/RFP reporter assay to confirm bonding between circRNA and miRNA. PmiR-RB-Report vectors (Jrdun bio, Shanghai, China) that contained both the firefly luciferase gene (hLuc+) and renilla luciferase gene (hRluc) were used in this study. We cloned the 3′ UTR sequence downstream of the hRluc cassette and constructed the miR-17-3p mimics from Jrdun bio (Shanghai, China) (forward: 5’-ACUGCAGUGAAGGCACUUGUAG-3’; reverse: 5’-ACAAGUGCCUUCACUGCAGUUU-3’). Each miRNA or negative control oligonucleotide was co-transfected with the pmiR-RB-Report vector with or without the 3′UTR sequence of circRNA_104670. Then, we measured the relative light units (RLU) for hRluc and hLuc+ using toolVeritas 9100-002 (Turner BioSystems, Sunnyvale, CA, USA). The value of hRluc was finally normalized to the corresponding hLuc+ value. A histogram was constructed to describe the experimental results.

We also performed the EGFP/RFP reporter assay to show that MMP-2 could bind to miRNA-17-3p. MiRNA-17-3p mimics and a negative control (NC) oligonucleotide (5’-UUGUACUACACAAAAGUACUG-3’) were constructed and then co-transfected with the pcDNA3 reporter vector, which contained the wild-type 3′UTR of MMP-2 or a mutant version of the 3′UTR of MMP-2. Finally, we examined the expression values of EGFP, which were normalized to the RFP values. A histogram was constructed to describe the results.

### RNA interference and transfection assay

We designed and synthesized small interfering RNAs (siRNAs) that targeted the back-splice junction of circRNA_104670 (si-circRNA_104670) and miR-17-3p (si- miRNA-17-3p) (Jrdun Bio, Shanghai, China). Based on the manufacturer’s protocol, the sequence of si-circRNA_104670 was 5’- AGAAGCAGGUUGAGGUGGUT-3’ (sense strand), 5’-ACCACCUCAACCUGCUUCUTT -3’ (antisense strand). For si-miRNA-17-3p, the sequence of the functional si-miRNA was CUACAAGUGCCUUCACUGCAGU. NP cells were transfected using Lipofectamine 2000 (Invitrogen, Carlsbad, CA, USA).

### NP cell viability assay

We carried out the MTT assay and cell flow cytometry for the NP cell viability assay. Transfected cells were obtained after 24 h. NP cells and transfected cells (si-circRNA_104670 and si-miRNA-17-3p) were collected and inoculated into a 96-hole plate with the appropriate number of cells per hole (100/ per hole) and then incubated with 5.0 mg/mL MTT. One hundred-fifty microliters of dimethyl sulfoxide was added to the sediments. Finally, we measured the absorbance using spectrophotometry at 490 nm and calculated the OD value. The normal NP cells and transfected cells (si-circRNA_104670 and si-miRNA-17-3p) were harvested after 24 h. The NP cells were stained with Annexin V and PI using an Annexin V-FITC/PI apoptosis detection kit (Jrdun Bio, Shanghai, China) before carrying out flow cytometry analysis (Genechem). We also performed cell cycle analysis by staining cells with propidium iodide using the Cycle TEST PLUS DNA Reagent Kit (Jrdun Bio, Shanghai, China). The NP cells were then observed and analyzed with FACScan.

### Western blotting and immunofluorescence analysis

Western blotting and immunofluorescence analyses of collagen II and MMP-2 were performed according to standard methods as the expression of MMP-2 was positively correlated with the IDD grade and the expression of collagen II was negatively correlated with the IDD grade^15–17^. Proteins were separated on a 10% sodium dodecyl sulfate-polyacrylamide gel and then transferred to polyvinylidene fluoride (PVDF) membranes. Then, the PVDF membranes were blocked in 5% milk for 30 minutes and incubated with primary antibody (MMP-2, dilution 1:200; Collagen Type II, dilution 1:200; GRAPH, dilution 1:1000; Santa Cruz Biotechnology, Inc.) for 12 h. We rinsed the blots several times with Tris-buffered saline containing 0.05% Tween-20 (TBST). The membranes were then incubated for 2 h with a goat anti-rabbit antibody (1:1000; Santa Cruz Biotechnology, Inc).

For immunofluorescence staining analysis, the cultured NP cells were blocked in blocking buffer for 1 h; then, the section was incubated with a primary antibody overnight at 4 °C (MMP-2, dilution 1:200; Collagen Type II, dilution 1:200; GRAPDH, dilution 1:1000; Santa Cruz Biotechnology, Inc.), followed by incubation with a secondary antibody (goat antirabbit) (1:100; Santa Cruz Biotechnology, Inc.). The section was rinsed three times in PBS for 5 min each, after which 4′, 6-diamidino-2- phenylindole (DAPI) was applied to counterstain the nuclei, and the cells were incubated for 5 min with a chromogen. Finally, the cells were examined using an inverted microscope.

### RNA inhibition animal grouping

All animal experiments were approved by the Institutional experimental animal Ethics Committee of Fudan University (Shanghai, China), and we performed the animal procedures strictly in accordance with the institutional and national guidelines to minimize suffering. All of the included C57BL/6J mice were purchased from Shanghai Slac Laboratory Animal Co. Ltd. (Songjiang, Shanghai, China) and housed in the approved animal care facility at the Center for Animal Experiments of Fudan University (Shanghai, China) in a 12:12—hour light—dark cycle environment. An animal model of interfering RNA expression (RNA inhibition) was developed by administering an efficient and safe vector for in vivo gene transfer, adeno-associated virus5-siRNA (AAV5- siRNA) (Jrdun Bio, Shanghai, China). All enrolled mice were divided into three groups: eight mice that received AAV5-normal saline were regarded as the control group; eight mice were used for circRNA_104670 inhibition, and eight mice were used for miRNA-17-3p and circRNA_104670 inhibition.

### Magnetic resonance imaging examination

Magnetic resonance (MR) images were taken at two time points (2 and 4 weeks) after the three groups of mice were enrolled in the study. The effects of the chosen gene on disc degeneration were evaluated using a Siemens Trio Tim 3.0T MR scanner (Siemens Medical Solutions, Erlangen, Germany) with a quadrature extremity coil receiver at the scheduled time (2 and 4 weeks). All mice in the three groups were anesthetized with a combination of 60 mg/kg ketamine hydrochloride and 10 mg/kg xylazine before the MR examination for the disc. The parameters of the MR scanner are described below: repetition time, 2200 ms; field of view, 60 mm × 60 mm; echo time, 66 ms; slice thickness, 0.8 mm; and in-plane resolution, 135.47 mm × 135.47 mm. The degree of disc generation was recorded and evaluated based on a modified Thompson classification, which specifically described the degree and area of signal intensity ranging from grades 1 to 4^18^. Two independent observers measured and recorded all of the data in a double-blind manner.

### Statistical analysis

Differences between the two groups were compared with two-tailed Student’s *t*-test for data that had a normal distribution and homogeneity of variance. Analysis of variance followed by the S-N-K post-hoc test were performed to calculate significant differences from multiple groups. All of the results are presented as the mean ± SEM. Fold-changes ⩾1.5 and *p* values < 0.05 in the microarray data were considered to be significantly different. All of the data analyses were performed using the SPSS software package (version 20.0, SPSS, Inc.).

## Results

### Profile of circRNA expression changed in degenerative tissues compared to that in normal NP tissues according to ceRNA analysis

**Fig. 1:**
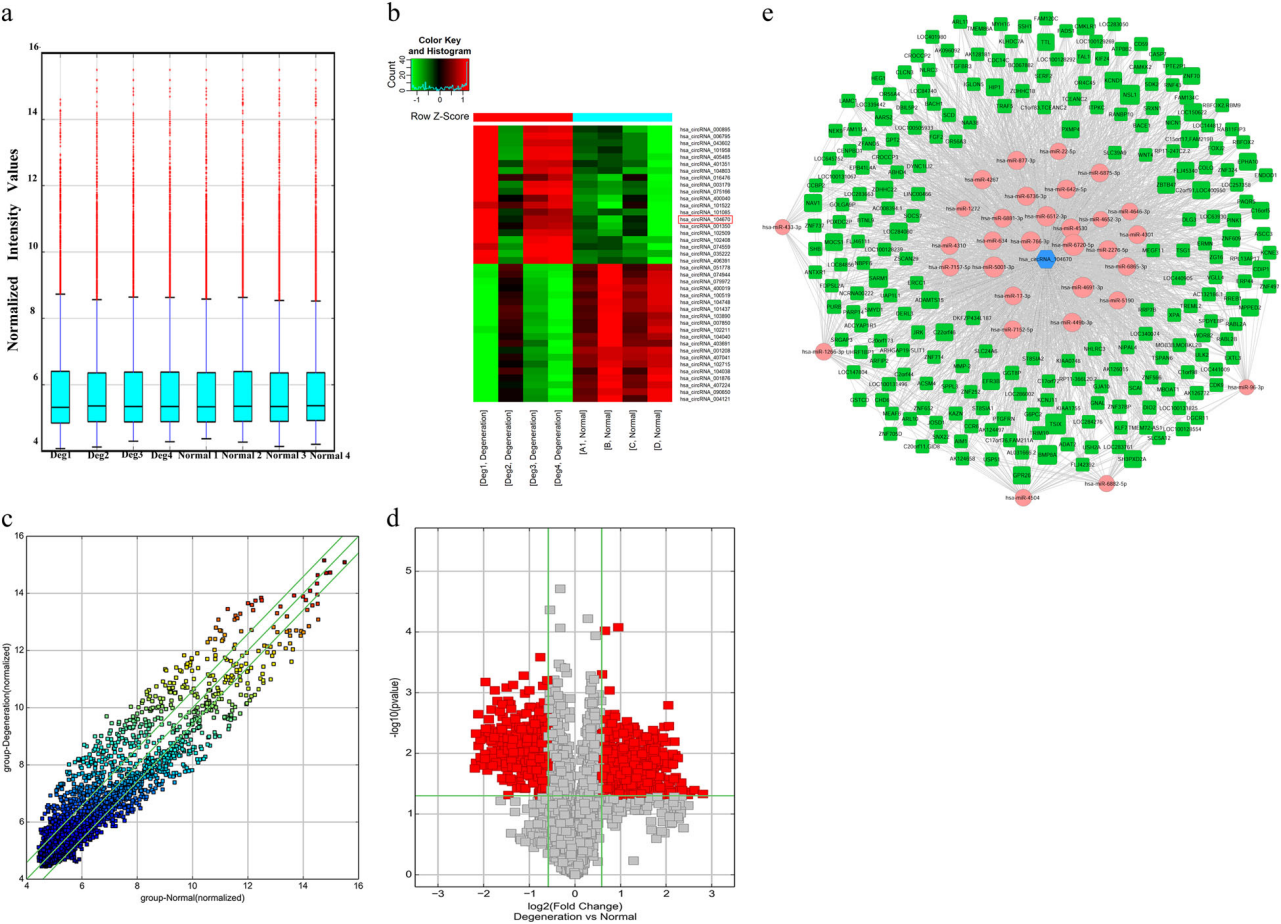
Differential expression of circRNAs in degenerative and normal NP tissues and ceRNA analysis for circRNA_104670. **a** The expression levels of circRNAs were recorded by a box plot as the mean ± standard deviation in the degenerative and normal NP groups. **b** Hierarchical clustering recorded the top 20 differentially expressed circRNAs between degenerative and normal NP tissues. The expression values are represented in different colors; red indicates high expression levels, and green indicates low expression levels. The red box indicates the selected circRNAs. **c** A scatter plot was used to evaluate the variation in circRNA expression. The values of the *X* and *Y* axes in the scatter plot correspond to the normalized signal values of the samples (log 2 scaled). The green lines are fold-change lines, the circRNAs above the top green and below the bottom line indicate greater than 1.5-fold changes between the two groups (*p* < 0.05). **d** Volcano plots were used to show differential expression of circRNAs. The vertical lines correspond to 1.5-fold up- and downregulation, with a *p* value of 0.05. The red point outside of the vertical lines in the plot shows the number of circRNAs that were differentially expressed with statistical significance between the two groups. **e** The interactions were specifically described basing on circRNA microarray and mRNA microarray data. In this network, 31 miRNAs were closely connected with circRNA_104670, while 26 miRNAs ranker higher. The hexagon at the center of the network indicates circRNA_104670, and the pink circles represent miRNAs that bound to this circRNA. The green quadrilaterals indicate target genes (miRNAs). The predicted interactions of ceRNA in the IDD process is circRNA_104670-miR-17-3p-MMP-2

A total of 792 circRNAs were differentially expressed in NP specimens between IDD and normal subjects based on the results of the microarray analysis; 428 were upregulated and 364 were downregulated (Fig. 1a, c, d). The top 20 upregulated circRNAs and top 20 downregulated circRNAs are listed in Fig. 1b. circRNA_104670 was selected for the verifying experiments as it was one of the highest upregulated circRNAs. The expression of circRNA_104670 in the IDD subjects was upregulated by nearly 4.5-fold.

To determine the proposed ceRNA mechanism, a network of circRNA-miRNA-mRNA was constructed using cytoscape, and their interactions were specifically described based on our circRN- miRNA-mRNA microarray data. Thirty-one miRNAs were closely bound to circRNA_104670, while 26 miRNAs ranker higher (Fig. 1e). MMP-2, a member of the matrix metalloproteinase family that plays an important role in the IDD process, is regarded as an miRNA target based on the results of the network. The predicted ceRNA mechanism was finally described in the IDD process: circRNA_104670 acts as a sponge or ceRNA for miRNA-17-3p, while MMP-2 is the target gene.

### Expression and diagnostic significance of circRNA_104670 and miR-17-3p

**Fig. 2:**
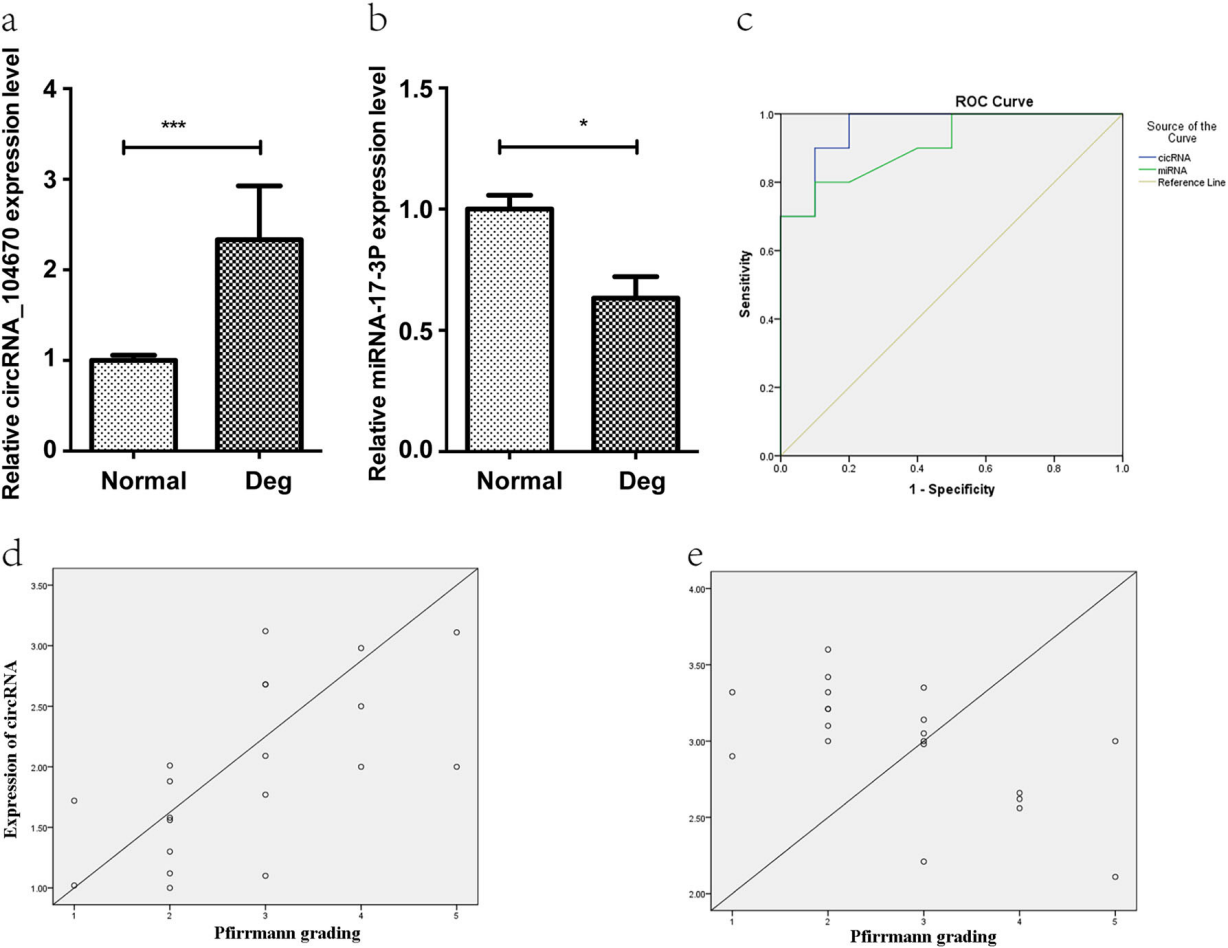
Expression and diagnostic significance of circRNA_104670 and miR-17-3p. **a**, **b** The degenerative group has higher expression of circRNA_104670 (*p* = 0.00) and lower expression of miR-17-3p (*p* < 0.05) compared with those of the normal group (p^***^ < 0.001; p^*^ < 0.05). **c** The ROC curve shows good diagnostic significance for circRNA_104670 and miR-17-3p, with AUC values of 0.96 and 0.91, respectively. **d**, **e** The circRNA_104670 and miR-17-3p expression levels are significantly correlated with the Pfirmman scores (*r* = 0.63; *p* = 0.00; *r* = −0.62; *p* = 0.00). ROC receiver-operating characteristic, AUC area under curve

Twenty NP tissues (ten from normal subjects and ten from IDD subjects) were used for quantitative real-time PCR to determine circRNA_104670 and miR-17-3p expression. The results showed that expression of circRNA_104670 in the degenerative group was much higher than that in the normal group (*p* = 0.00), while the expression of miR-17-3p in the degenerative group was lower compared with that in the normal group (*p* < 0.05) (Fig. 2a, b). The ROC curve showed that circRNA_104670 and miR-17-3p had good diagnostic significance for IDD (AUC circRNA_104670 = 0.96; AUC miRNA-17-3p = 0.91) (Fig. 2c). A significant correlation was detected between the Pfirrmann grade and expression of the selected RNAs; circRNA_104670 expression was positively correlated with the Pfirmann grade (*r* = 0.63; *p* = 0.00), while the expression of miR-17-3p was negatively correlated with the Pfirrmann grade (Fig. 2d) (*r* = −0.62; *p* = 0.00) (Fig. 2e).

### CircRNA_104670 acts as a sponge for miR-17-3p, and MMP-2 is directly targeted by miR-17-3p

**Fig. 3:**
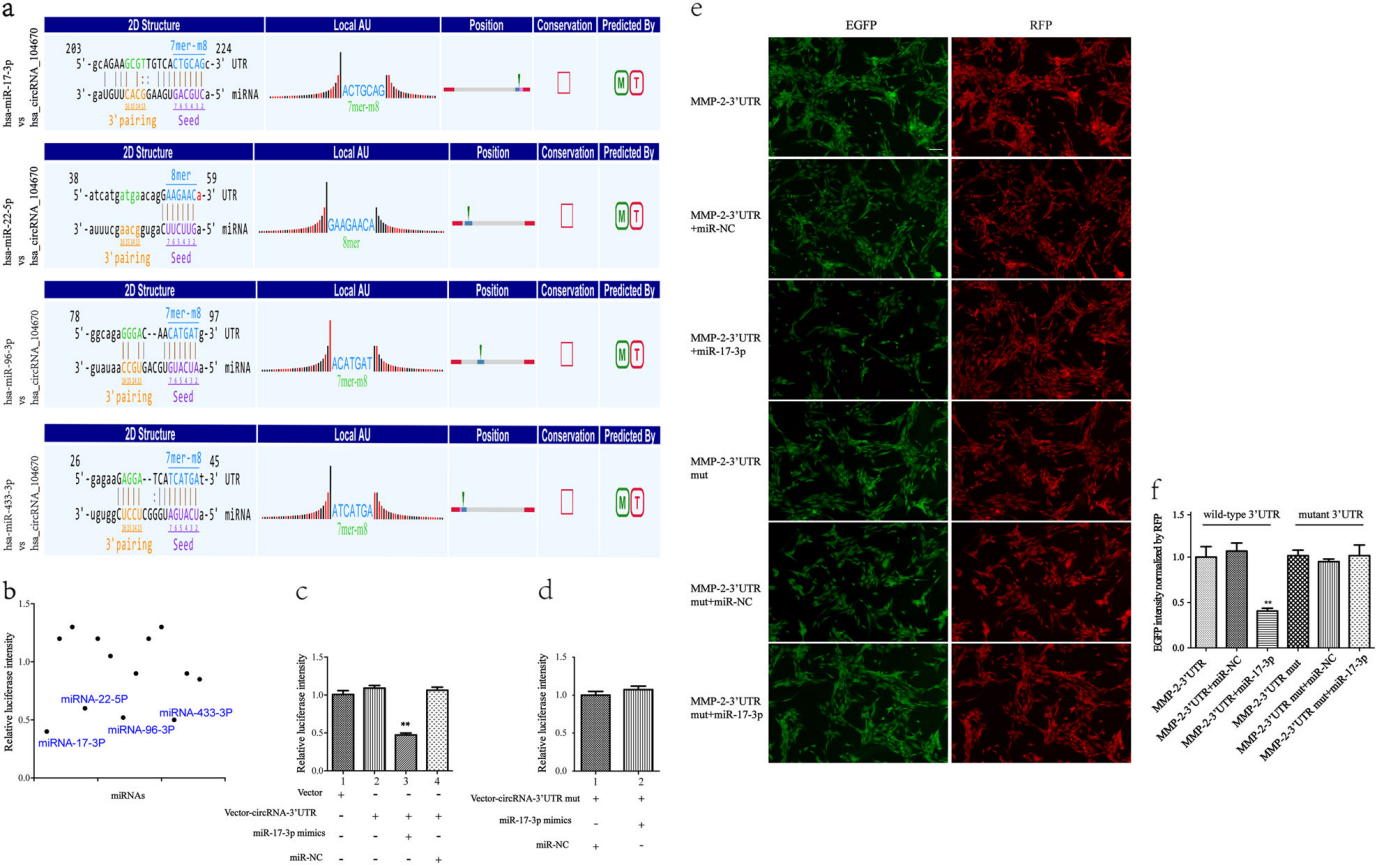
CircRNA 104670 serves as a sponge for the miR-17-3p and MMP-2 is directly targeted by miR-17-3p. **a** The binding region of miRNAs in circRNA_104670 3′UTR are shown. **b** A luciferase reporter assay confirmed that miR-17-3p, miR-22-5p, miR-96-3p, and miR-433-3p reduce the luciferase intensity by more than 40%. **c**, **d** A pmiR-RB-Report vector with or without the 3′UTR sequence of circRNA_104670 was co-transfected with miR-17-3p or a negative control oligonucleotide; RLU of hRluc and hLuc+ were constructed, and the hRluc values were normalized to the corresponding hLuc+ values, **p < 0.01. **e**, **f** NP cells were co-transfected with an EGFP reporter plasmid (containing 3′UTR of MMP-2) or the mutant vector (containing mutant 3′UTR of MMP-2) the pDsRed-C1 plasmid either alone or in combination with a miR-17-3p mimic. An F-4500 fluorescence spectrophotometer was used to analyze the EGFP and RFP levels. Scale bar, 40 μm, **p < 0.01

Based on ceRNA analysis, circRNA_104670 was able to directly bind to 31 miRNAs; however, four miRNAs (miRNA-17-3p, miRNA-22-5p, miRNA-96-3p, miRNA-433-3p) were finally selected because they ranked highly in correspondence with the positions of the putative binding sites in the 3′-untranslated region (3′UTR) of circRNA_104670 (Fig. 3a). The luciferase intensity was measured after each miRNA mimic and luciferase reporter was co-transfected into NP cells. The results showed that the four miRNAs mentioned above led to luciferase intensity reductions of more than 40%, while miR-17-3p led to the largest intensity reduction (over 60%) (Fig. 3b, c). To further confirm that miR-17-3p was able to bind to circRNA_10467, we mutated the miRNA response elements (MREs) of miR-17-3p in the luciferase reporter, and we observed that co-transfection of miR-17-3p mimics, and the mutated luciferase reporter had no significant difference on the luciferase activity (*p* > 0.05) (Fig. 3d).

The EGFP/RFP reporter assay was conducted to examine whether MMP-2 could directly bind to miR-17-3p. MiR-17-3p mimics with wide-type reporter plasmids or mutated vectors were co-transfected into NP cells. The EGFP intensity values were normalized to that of RFP. The results confirmed that co-transfection of miR-17-3p mimics and the wide-type reporter plasmid into NP cells strongly reduced EGFP expression (*p* < 0.01), while co-transfection of miR-17-3p mimics and mutated vectors in NP cells did not affect EGFP expression (*p* > 0.05) (Fig. 3e, f).

### CircRNA_104670 promotes apoptosis and inhibits proliferation of NP cells

**Fig. 4:**
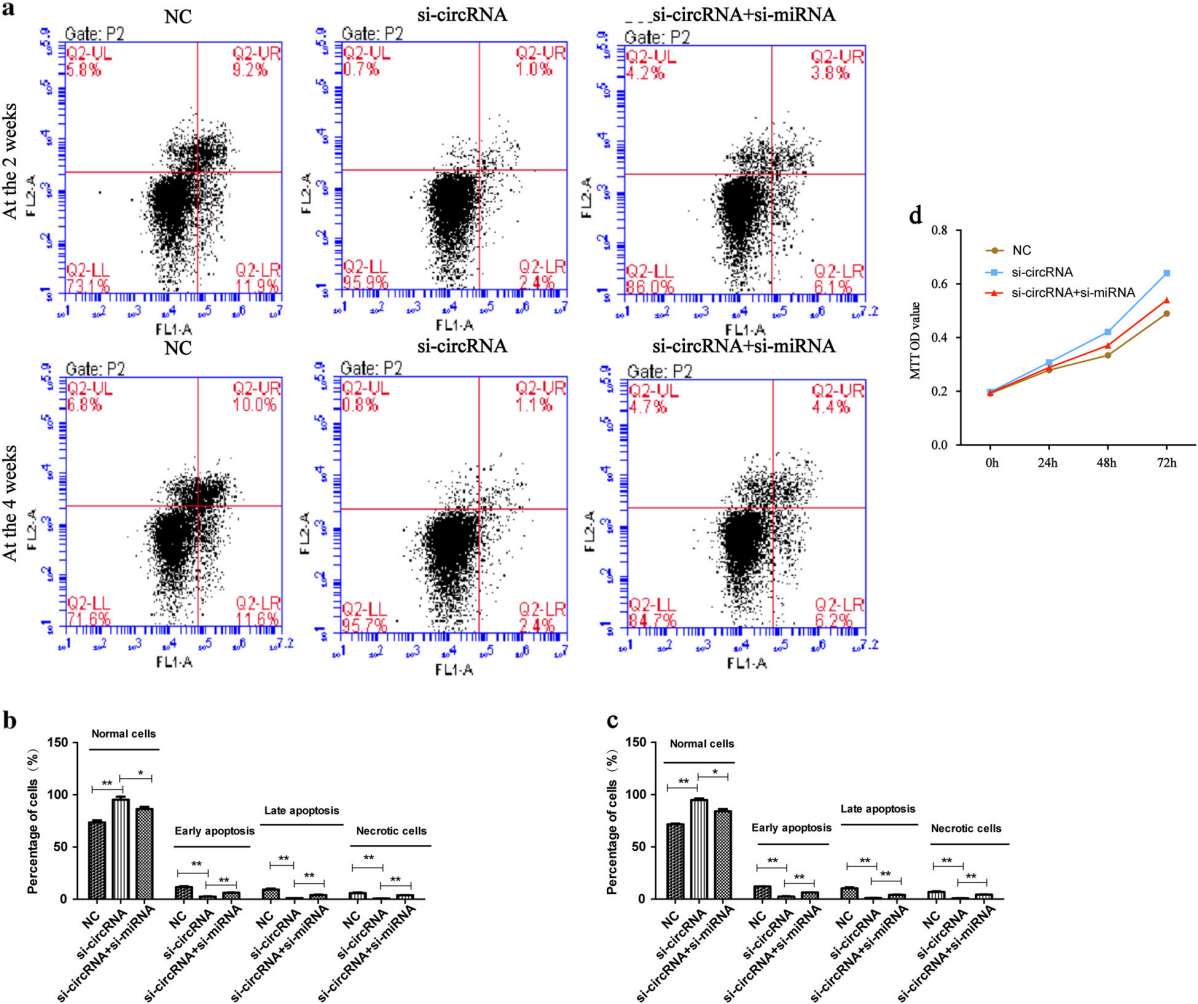
circRNA_104670 promotes apoptosis and inhibits the proliferation of NP cells. **a**–**c** Cell flow cytometry was carried out to explore the NP cell viability at 2 and 4 weeks after the si-miRNAs were co-transfected into NP cells. The plots shown on the button left indicate normal NP cells, the plots shown on the bottom right indicate early apoptotic NP cells, the plots shown on the top right indicate late apoptotic NP cells and the plots shown on the top left indicate necrotic NP cells. **d** An MTT assay was performed in NP cells at 0, 24, 48, and 72 h after transfections. *p < 0.05; **p < 0.01

siRNAs that targeted the back-splice junction of circRNA_104670 (si-circRNA_104670), miR-17-3p (si-miRNA-17-3p) were designed and synthesized. NP cells were randomly divided into three groups: negative control (NC) groups with NP cells, NP cells with si-circRNA_104670, and NP cells with si-circRNA_104670 and si-miRNA-17-3p. Cell flow cytometry was carried out to evaluate the NP cell viability at 2 and 4 weeks after the si-miRNAs were co-transfected into NP cells, and the MTT assay was used to examine the proliferation of NP cells at the 0, 24, 48, and 72 h after transfection. The cell flow cytometry results at 2 and 4 weeks showed that interfering with the circRNA_104670 inhibited apoptosis of NP cells (*p* < 0.01), while interfering with both the circRNA_104670 and miR-17-3p reduced this inhibition (*p* < 0.01) (Fig. 4a, b, c). The results of the MTT assay demonstrated that interfering with circRNA_104670 promoted NP cell proliferation (*p* < 0.05), while interfering with both circRNA_104670 and miR-17-3p reduced this proliferation (*p* < 0.05) (Fig. 4d).

### circRNA_104670 inhibits the expression of collagen II and promotes the expression of MMP-2

**Fig. 5:**
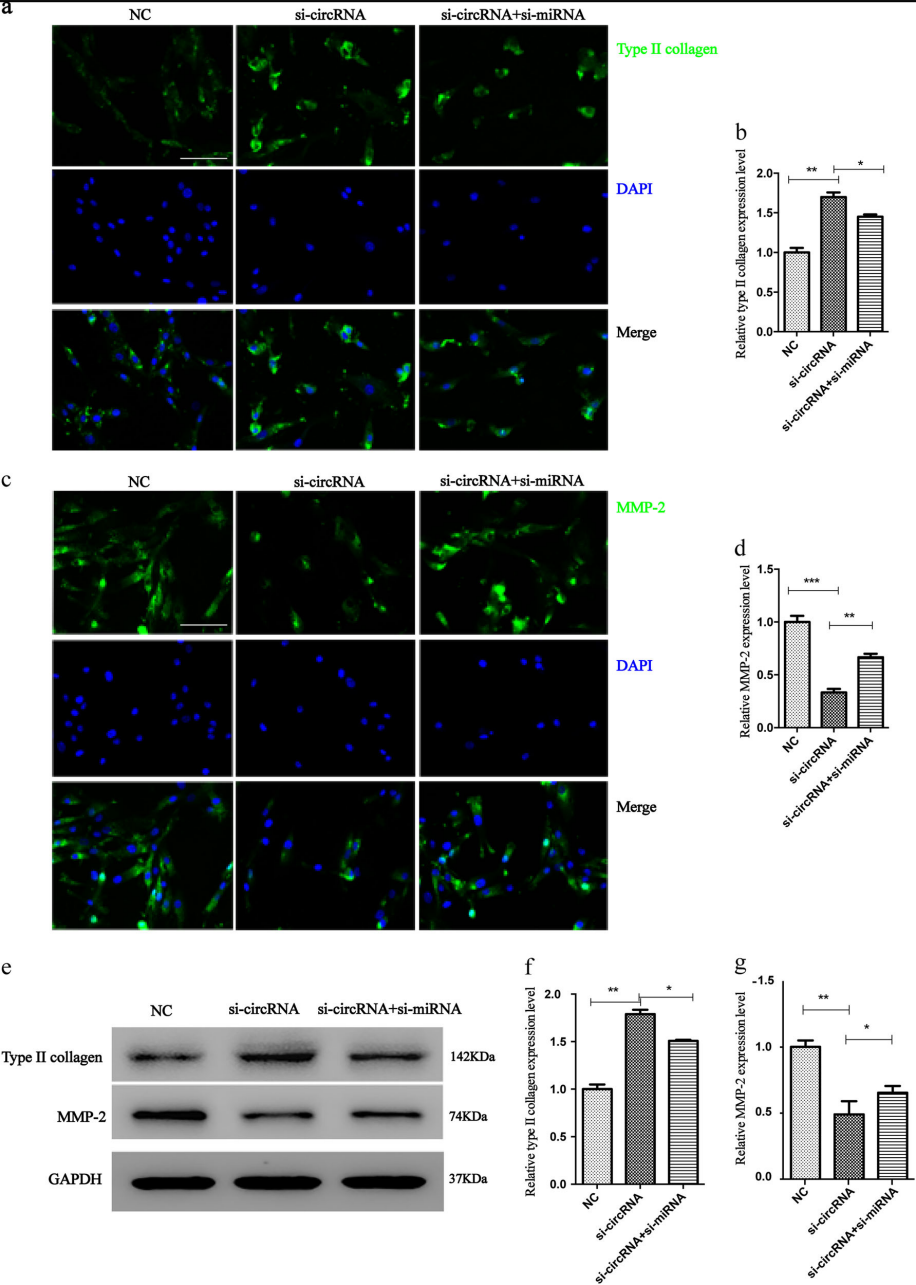
circRNA_104670 promotes the expression of MMP-2 and inhibits the expression of collagen II. **a**, **b** Immunofluorescence staining for collagen II after transfection with si-circRNA_104670 or co-transfection with si-circRNA_104670 and si-miRNA-17-3p, scale bar, 40 μm. **c**, **d** Immunofluorescence staining for MMP-2 after transfection with si-circRNA_104670 or co-transfection with si-circRNA_104670 and si-miRNA-17-3p, scale bar, 40 μm. **e**–**g** Collagen II and MMP-2 protein expression levels were analyzed by Western blotting. GAPDH was used as a loading control. The presented values are the means ± SD. of three different preparations, *p < 0.05; **p < 0.01; ***p < 0.001

To analyze the effects of circRNA_104670 on ECM degradation, we examined the effects of circRNA_104670 and miR-17-3p knockdown in NP cells. Western blotting and immunofluorescence analyses were performed according to the standard methods to explore the expression of collagen II and MMP-2 in three groups of NP cells: NC groups with NP cells, NP cells with si-circRNA_104670, and NP cells with si-circRNA_104670 and si-miRNA-17-3p. The Western blotting immunofluorescence analyses results showed that interfering with circRNA_104670 promoted the expression of collagen II (*p* < 0.01), while this effect was reduced by interfering with both circRNA_104670 and miR-17-3p (*p* < 0.05) (Fig. 5a, b, e, f). It was also suggested that interfering with circRNA_104670 inhibited the expression of MMP-2 (*p* < 0.01), while this inhibition was reduced by interfering with both circRNA_104670 and miR-17-3p (*p* < 0.05) (Fig. 5c, d, e, g).

### MRI indicates that circRNA_104670 accelerates intervertebral disc degeneration in gene inhibition mice

**Fig. 6:**
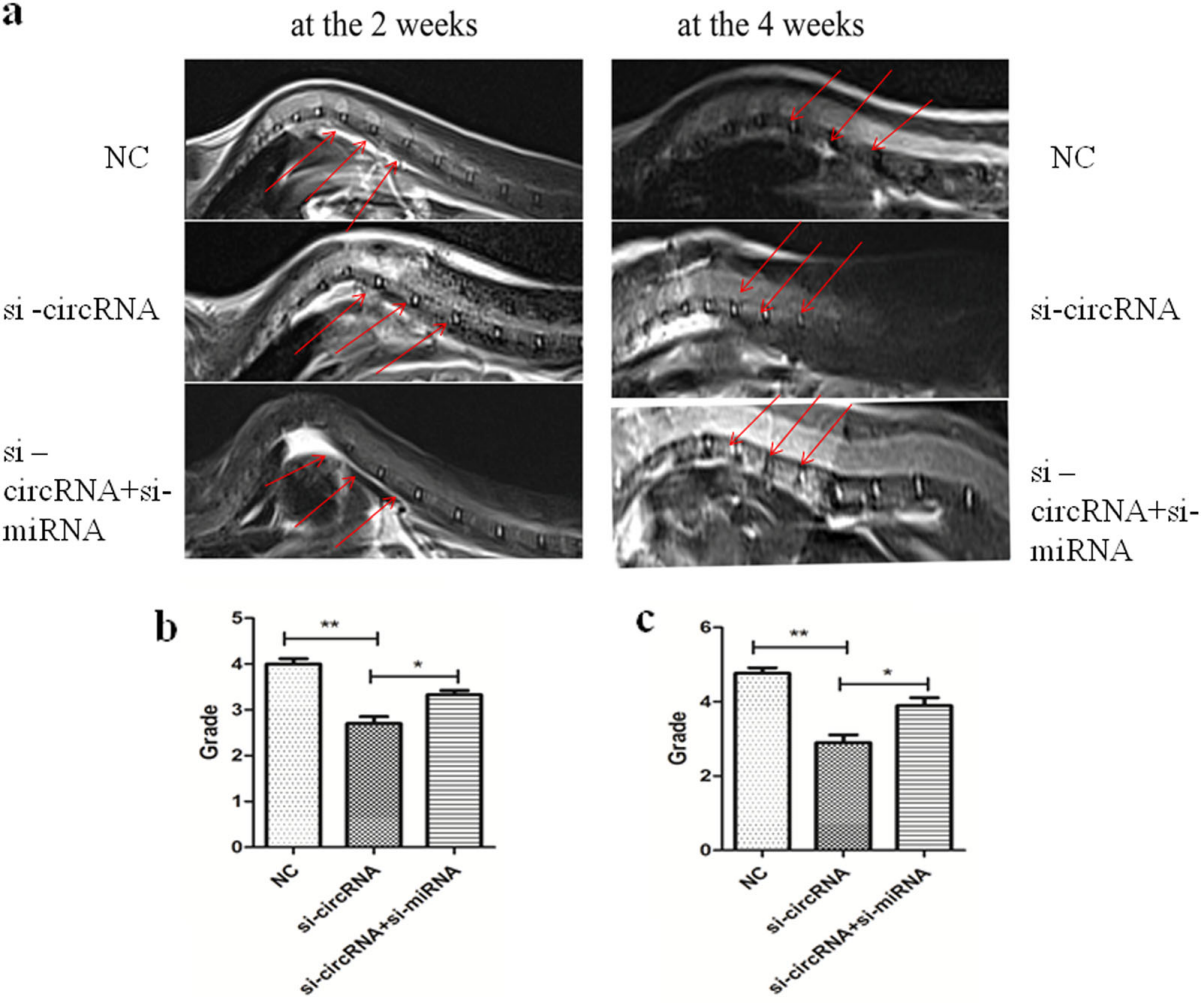
MRI evaluation in circRNA_104670 or circRNA_104670 with miR-17-3p gene inhibition mice at the 2 and 4 weeks. **a** Three representative T2-weighted magnetic resonance images from each group (the original in-plane resolution is 135.47 mm × 135.47 mm) at 2 (left panels) and 4 (right panels) weeks after gene inhibition of mice are shown. **b**, **c** Quantitative data of the MRI grades in three groups (*n* = 8/group) at 2 (**b**) and 4 (**c**) weeks after the birth of the mice are shown. *p < 0.05, **p < 0.01

Mice with circRNA_104670 or circRNA_104670 with miR-17-3p inhibition were obtained. All mice were divided into three groups with eight mice each: the control group, circRNA_104670 gene inhibition mice (*n* = 8); miR-17-3p, and circRNA_104670 double gene inhibition mice (*n* = 8). The IDD grade was recorded for three selected levels at 2 and 4 weeks after birth based on the modified Thompson classification, which describes the degree and area of signal intensity from grades 1 to 4^14^. The results showed that no significant differences in the IDD grade were detected for the three groups 2 weeks after birth (*p* > 0.05). It was also shown that circRNA_104670 gene inhibition mice had a lower IDD grade compared with control group mice (*p* < 0.01), while circRNA_104670 and miR-17-3p double gene inhibition mice had a higher IDD grade compared with circRNA_104670 gene interfering mice (*p* < 0.05) (Fig. 6). These results suggested that circRNA_104670 accelerated the IDD process, while miR-17-3p inhibited the IDD process induced by circRNA_104670. These results were in agreement with the ceRNA theory verified by the in vitro experiments.

## Discussion

NcRNAs are composed of small nuclear RNA (*sn*RNA*s*), small nucleolar RNA (snoRNA), long ncRNAs (lncRNAs), small ncRNAs (miRNAs), and the newly defined circRNAs in terms of length and structure, and the number of ncRNAs have been shown to play fundamental roles in some aspects of organisms^19–21^. CircRNAs have become the focus of a large amount of research in recent years. CircRNAs are defined as a naturally existing family of ncRNAs, and they are highly represented in the eukaryotic transcriptome^22^. CircRNAs are characterized by a covalent loop configuration without 50 to 30 polarity or a polyadenylated tail, which are different from the conventional linear RNAs, which end with 50 caps and a 30 tail; these intrinsic characteristics may have resulted in an underestimation of the roles of circRNAs in previous polyadenylated transcriptome analyses^23,24^. Several recent studies confirmed that circRNAs could be formed by exonic or intronic transcripts through non-linear reverse splicing or gene rearrangement. Both exonic or intronic transcripts of circRNAs play important roles in regulating gene expression^25–28^. It has been confirmed that circRNAs can absorb miRNA and overcome the original repression on the miRNA-targeted gene by acting as a post-transcriptional regulator (miRNA sponge); this type of RNA is defined as competing endogenous RNA (ceRNA) in the cytoplasm^29^. A study detected 1903 circRNAs in mouse tissues, including brains, fetal head and embryonic stem cells, while 83 of the included circRNAs were also found in human tissues ^30^. Bachmayr-Heyda et al.^31^ detected and compared the expression abundance of circRNAs in 13 human tissues, and the results showed that the most abundant tissue with circRNAs expression was the brain, while the lowest was the muscle.

Aging of NP cells is likely the main cause of IDD. As in many aspects of general biology, RNAs participate in the regulation of the aging process and age-related diseases^32,33^. The transcribed RNA profiles of cells and tissues change during aging. Several methods have been used to connect the aging process with RNA. Changes in the oxidative stress response and, specifically, the mitochondrial electron transport chain may contribute to the linkage of the aging process and RNA molecules; gene mutations associated with accelerated aging are also believed to affect RNA by strongly interacting with DNA repair pathways. Some evidence has also confirmed that the altered use of RNAs via protein translation mediated, at least in part, by the mTOR pathway may strongly contribute to age-related phenotypes. Finally, age-related neurodegenerative conditions may provide the aging process with access to the RNAs^34–38^. CircRNAs are also closely associated with the aging process. Gruner et al.^39^ carried out RNA sequencing in young (1-month-old) and aged (22-month-old) hippocampus, cortex, and heart samples and detected 6791 distinct circRNAs in these samples, including 675 novel circRNAs. Another study confirmed that functional disturbance of circRNA-miRNA-mRNA regulatory systems may represent another level of epigenetic control over pathogenic gene expression pathways in the human central nervous system, which are targeted by the sporadic Alzheimer’s disease process^40^. These findings suggest that the biological process of nervous system aging may be regulated by a large number of circRNA molecules. CircRNAs expressed in the osteoarthritis and normal cartilage were compared and analyzed, and the results showed that a number of 71 circRNAs were found to be differentially expressed. By contrast, a selected circRNA (circRNA_100086) regulated MMP13 expression by functioning as a ceRNA and participating in the chondrocyte ECM degradation process^41^.

In the present study, the microarray analysis results showed that 792 circRNAs were differentially expressed in NP specimens from IDD and normal subjects, and 428 circRNAs were upregulated and 364 circRNAs were downregulated. circRNA_104670 was selected as the target circRNA because it had the largest change in expression in the IDD tissues. It was confirmed that the circRNAs also acts as a ceRNA in the regulatory process of IDD. A network of circRNA-miRNA-mRNA was also constructed, indicating the potential associations between circRNAs and their target genes. According to the results of circRNA microarray analysis, circRNA_104670 harbors miRNA-binding sites, including for miR-17-3p, miR-22-5p, miR-96-3p, and miR-433-3p, among others. To determine the potential interaction between circRNA_104670 and miRNA-17-3p, a luciferase assay was performed, and the luciferase intensity was found to be reduced by 60% compared with that of NP cells co-transfected with the mimic control and reporter vector. We then mutated the miRNA MREs of miR-17 3p in the luciferase reporter, and it was observed that co-transfection of the miR-17-3p mimics and mutated luciferase reporter had no significant difference on the luciferase activity (*p* > 0.05). The results of the luciferase assay showed that circRNA_104670 was able to directly bind to miR-17-3p. An EGFP/RFP reporter assay was also performed to determine whether MMP-2 was able to directly bind to miRNA-17-3p. It was observed that co-transfection of miR-17-3p mimics and wide-type reporter plasmid into NP cells strongly reduced EGFP expression (*p* < 0.01), while co-transfection of miR-17-3p mimics and the mutated vector into NP cells did not affect EGFP expression. The results of the EGFP/RFP reporter assay showed that miR-17-3p was also able to bind MMP-2 and efficiently inhibit translation of the chimeric transcript. Thus, we confirmed that circRNA_104670 functions as a decoy to regulate MMP-2 expression by acting as a ce-RNA of miR-17-3p. The two referenced RNAs showed good diagnostic significance for IDD based on the results of the AUC curve; the expression of circRNA_104670 was positively correlated with the IDD grade, while the expression of miR-17-3p was a negatively correlated with the IDD grade (*p* < 0.05). The above results may provide a better target for IDD therapy compared to conventional methods.

MMPs appear to be important regulators of tissue remodeling and repair, but the overexpression of MMPs in IVD could lead to the degradation of all of the ECM components that exist within the IVD, ultimately resulting in IDD. Collagen type II was observed to be highly expressed in juvenile NP tissues, while this expression decreased with biological aging. It is therefore believed that the expression of MMP-2 is positively correlated with the IDD grade, and that the expression of collagen II is negatively correlated with the IDD grade^42-44^. The Western blotting and immunofluorescence analyses results showed that interfering with circRNA_10467 inhibited the expression of MMP-2 and promoted the expression collagen type II, while blocking miR-17-3p promoted MMP-2 expression and inhibited collagen type II expression. Thus, these findings demonstrate that circRNA_104670 accelerates the IDD process by acting as a sponge for miR-17-3p. The IDD process is always accompanied by large number of apoptotic and necrotic NP cells. It is vital to examine the biological activity of NP cells as IDD proceeds. The results of the MTT assay and cell flow cytometry showed that interfering with circRNA_104670 decreased the amount of apoptotic NP cells, which were previously considered to be the cause of IDD, while interfering with miR-17-3p increased the amount of apoptotic NP cells. MR images of gene inhibition mice at 2 and 4 weeks showed that the interfering with circRNA_104670 inhibited the degeneration of the IVD in mice. Therefore, interfering with miR-17-3p promoted IDD, consistent with the results of the in vitro experiment.

In conclusion, the present study uncovers and validates IDD-specific circRNA transcriptome profiles, in which circRNA_104670 is upregulated in human IDD tissues and regulates mRNA (MMP-2) by directly sponging miRNA-17-3p (ceRNA). These data may provide a novel potential therapeutic target for patients with IDD.
